# Alexander's Law During High-Speed, Yaw-Axis Rotation: Adaptation or Saturation?

**DOI:** 10.3389/fneur.2020.604502

**Published:** 2020-11-23

**Authors:** Claudia Lädrach, David S. Zee, Thomas Wyss, Wilhelm Wimmer, Athanasia Korda, Cinzia Salmina, Marco D. Caversaccio, Georgios Mantokoudis

**Affiliations:** ^1^Department of Otorhinolaryngology, Head and Neck Surgery, Inselspital Bern, Bern, Switzerland; ^2^Department of Neurology, Johns Hopkins University School of Medicine, Baltimore, MD, United States; ^3^Hearing Research Laboratory, ARTORG Center, University of Bern, Bern, Switzerland

**Keywords:** Alexander's law, nystagmus, vestibulo-ocluar reflex, gaze-dependent nystagmus, eye-velocity-to-position integrator

## Abstract

**Objective:** Alexander's law (AL) states the intensity of nystagmus increases when gaze is toward the direction of the quick phase. What might be its cause? A gaze-holding neural integrator (NI) that becomes imperfect as the result of an adaptive process, or saturation in the discharge of neurons in the vestibular nuclei?

**Methods:** We induced nystagmus in normal subjects using a rapid chair acceleration around the yaw (vertical) axis to a constant velocity of 200°/second [s] and then, 90 s later, a sudden stop to induce post-rotatory nystagmus (PRN). Subjects alternated gaze every 2 s between flashing LEDs (right/left or up/down). We calculated the change in slow-phase velocity (ΔSPV) between right and left gaze when the lateral semicircular canals (SCC) were primarily stimulated (head upright) or, with the head tilted to the side, stimulating the vertical and lateral SCC together.

**Results:** During PRN AL occurred for horizontal eye movements with the head upright and for both horizontal and vertical components of eye movements with the head tilted. AL was apparent within just a few seconds of the chair stopping when peak SPV of PRN was reached. When slow-phase velocity of PRN faded into the range of 6–18°/s AL could no longer be demonstrated.

**Conclusions:** Our results support the idea that AL is produced by asymmetrical responses within the vestibular nuclei impairing the NI, and not by an adaptive response that develops over time. AL was related to the predicted plane of eye rotations in the orbit based on the pattern of SCC activation.

## Introduction

Alexander's law (AL) is commonly shown by patients with spontaneous nystagmus (SN) due to a vestibular imbalance. The nystagmus is more intense, with a higher velocity of the slow phase, when patients gaze in the direction of the quick phase (1). Two main hypotheses have been invoked to account for AL. First, AL has been attributed to a slowly developing, adaptive mechanism that lessens the slow-phase velocity of a pathological SN when gaze is in the direction of the slow phase (2). According to this hypothesis, the neural circuit for eccentric gaze holding is “purposefully” impaired, causing the eyes to drift centripetally so that in one direction of gaze a bias from the centripetal drift opposes and diminishes the SN. Normally eccentric gaze is held steady by a neural network called the ocular motor neural integrator (NI) (3). How well the NI performs is judged by its time constant (TcNI, [seconds, s]) of decay when an input is no longer present. The more perfect the integrator the higher the value of the time constant being in the range of 20–40 s (4). When the TcNI is low, making the NI “leaky,” the eyes drift centripetally on eccentric gaze so when a patient with SN looks in the direction of the slow phase, the SN is reduced, gaze is better stabilized, images move less on the retina, and vision is improved (2, 5).

Alternative hypotheses suggest that AL arises as an epiphenomenon from a non-linear interaction in the vestibular nuclei (6, 7) or in the ocular motor nuclei (8), when processing activity for the vestibulo-ocular reflex (VOR). One difficulty in arriving at a more definitive explanation for AL are the different types of stimuli (caloric, one ear vs. both ears stimulation, total body vs. head only rotations), stimulus characteristics (frequency, intensity, duration, stimulus profiles) and subject populations (healthy vs. pathological) that have been used to investigate AL. The aim of this study was to buttress one or the other of these hypotheses. We examined the influences on AL of a high-speed, constant-velocity, chair rotation in *healthy subjects* to induce a prolonged nystagmus as a surrogate for SN in patients. We particularly focused on (1) the onset of AL relative to the onset of nystagmus, (2) the effect of the magnitude of the SPV of nystagmus on AL, and (3) the effect of stimulating different patterns of the semicircular canals on AL.

## Materials and Methods

### Subjects

We tested nine healthy subjects from 24 to 54 years of age (mean 33.2 years, SD ± 10.9 years), four women and five men. Subjects had no history of vestibular, ocular, or neurologic dysfunction, and had normal vision and normal ocular motor function. All included subjects had a normal rotational velocity step test including a normal time constant. Subjects had no spontaneous nystagmus (SN) at rest.

### Rotational Chair Stimuli

We used a yaw-axis, rotational chair paradigm (Mini Torque, DIFRA, France) in complete darkness with acceleration to a constant velocity of 200°/s within 1 s. We used the same pattern of alternating rotational directions for all subjects to compare systematically right/left and up/down gaze. The rotation period lasted 90 s and was followed by a sudden deceleration of 200°/s^2^ to an abrupt stop.

**Figure 1:**
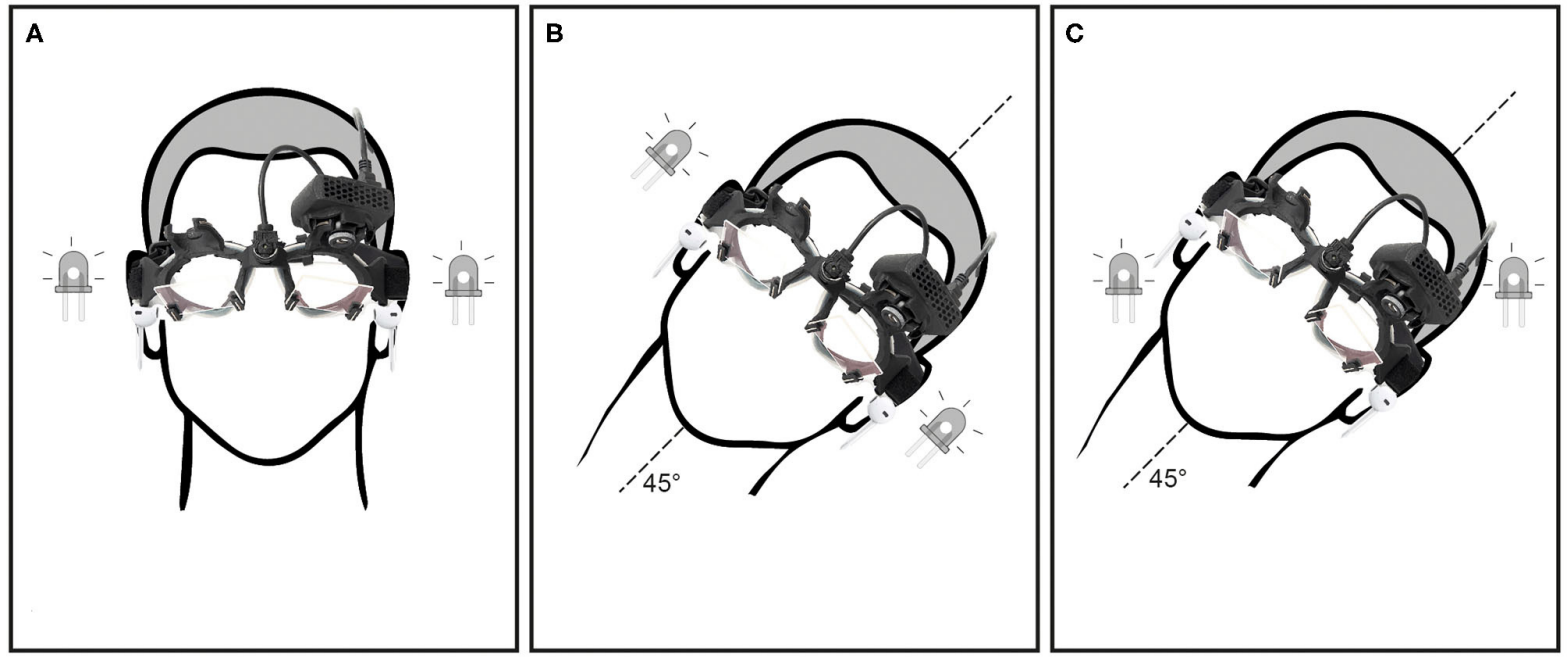
Showing different head and LED positions: **(A)** head upright, LED positioned for horizontal eye movements in the orbit, **(B)** head tilt 45° to the left, LED positioned for horizontal eye movements in the orbit and **(C)** head tilt 45° to the left, LED positioned for oblique (combined horizontal and vertical) eye movements in the orbit.

**Figure 2:**
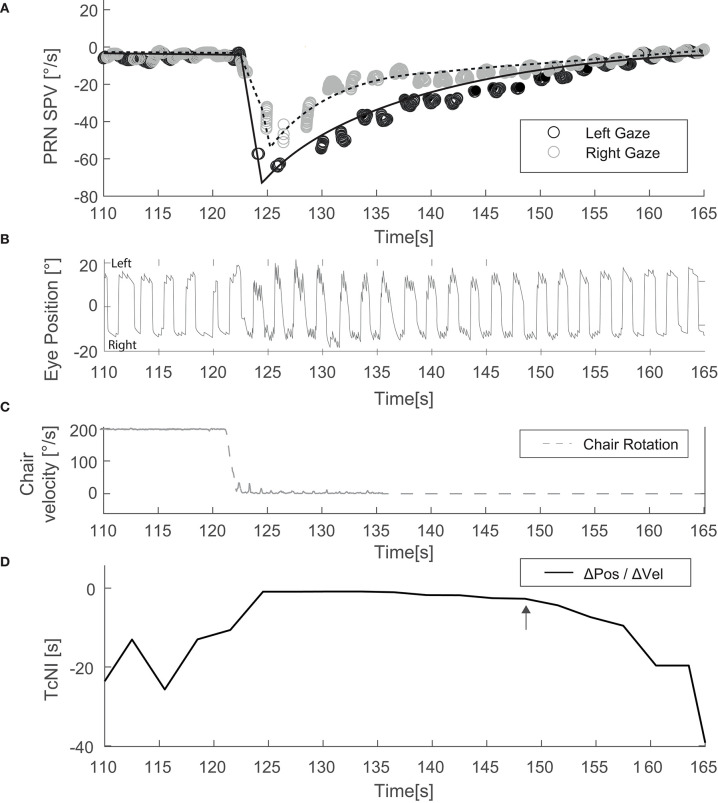
Example of an experiment with the head upright and alternating horizontal gaze (Condition Figure 1A). **(A)** Depicts SPV over time just before and after the chair has stopped. **(B)** Illustrates the corresponding eye position data. **(C)** Shows the rotational stimulus used (chair velocity). **(D)** Shows TcNI as a function of time. The arrow indicates the estimated point when the TcNI began to recover toward normal.

All subjects were tested in two head positions, (1) upright primary head position with head pitched 30° downward, maximally stimulating the lateral SCCs (Figure 1A) and (2) head rolled (tilted) 45° to the left, stimulating both the lateral and vertical SCCs (Figures 1B,C). One other subject was tested with the head upright (Figure 1A) at two rotational velocities (100 and 200°/s).

### Video-Oculography (VOG)

We recorded horizontal and vertical eye movements at a frame rate of 200 Hz using the video-oculography (VOG) device with a single infrared camera mounted on a goggle frame (Eyeseecam, EyeSeeTec, Munich), therefore tracking the movement of one eye, leaving the other covered (avoiding double vision or vergence). The VOG device was calibrated for horizontal and vertical eye positions using its built-in calibration system with laser projections on the wall at 1.5 m distance.

### Flashing Light Targets

To compare AL with different target locations on separate trials subjects were asked to look at LEDs positioned in the goggles to the right and left (±18°, Figures 1A,B) or diagonally (up and down ±13°, Figure 1C), during and after rotation. The LEDs were flashed every second for 20 ms. Subjects were cued every second to alternate the direction of their gaze to the other LED by a beep heard through headphones.

### SPV and Alexander's Law

The slow-phase velocity (SPV) of the nystagmus was calculated using custom MATLAB software scripts by differentiating eye positions (EPos) after a de-saccading procedure using median filters. Data quality was checked and outliers including remaining saccades removed. Data points around the center gaze position (−10 *to* +10°) were excluded from analysis. SPV>100°/s or slow phases in the wrong direction were considered outliers. We recorded and analyzed the PRN and the nystagmus at rest prior to stimulation. We calculated the time constant of the VOR (TcVOR) for each gaze position. The decay of SPV during PRN was fitted with a negative exponential curve function y = A^*^e^∧^[b^*^(−*t*)] to calculate the peak SPV (A) and TcVOR (b) over time (t). TcVOR indicated the time when SPV declined to 63% of its peak value. At each gaze interval of 2 s, the instantaneous difference between the opposing eye positions at peak SPV (ΔEPos = left EPos – right EPos), and the instantaneous difference in SPV between right and left gaze (ΔSPV = left gaze SPV – right gaze SPV) were calculated. The corresponding values of the time constant (TcNI), which reflects the fidelity of the NI for holding positions of gaze, was calculated as follows:

$$\begin{array}{l} \text{TcNI}=\Delta \text{EPos}/\Delta \text{SPV } \end{array}$$

We also determined the time between when the chair stopped, and the SPV of PRN reached its peak. We estimated by eye when the TcNI began to return to its normal value by choosing the time point during PRN at a clear inflection point (e.g., Figure 2D, arrow) and its corresponding mean SPV.

### Statistics

Differences in outcome measures were estimated using separate linear mixed-effects models for each stimulation condition (head upright vs. head tilted) and for horizontal and vertical eye movements separately. We evaluated SPV at peak velocity (A) and the TcVOR (b) separately as well.

We used gaze direction (right vs. left or up vs. down) as fixed effects and a subject-level random effect to account for paired measurements. To compare horizontal ΔSPV between the two stimulations, we included the test condition (head upright vs. head tilted) interacting with the gaze direction. We used general linear hypothesis testing with two-tailed tests and Holm correction for *post-hoc* comparisons among the test conditions (9). A significance level of 0.05 was used for all comparisons. The statistical analysis was performed with the R environment (v3.4, R Core Team) (10).

## Results

We excluded trial runs when datasets did not meet pre-defined quality criteria. The main reasons for excluding data were poor VOG eye tracking due to eyelid artifacts or blinking, and inability to maintain eye position at the locations of interest. For the assessment of AL in horizontal eye positions, eight datasets fulfilled the inclusion criteria for lateral SCC stimulation, and seven for combined stimulation. For vertical eye positions, six data sets fulfilled the inclusion criteria.

Mean TcNI for eccentric right and left gaze before rotation was 21 s (±8.2). Figure 2 shows an example for horizontal SPV of PRN at both right and left gaze in the head upright position (Figure 1, condition A). There is a stronger PRN in left gaze than in right gaze, thus following AL. Panel D in Figure 2 depicts how the TcNI varied over time. AL appeared virtually immediately, at least within the resolution of our measurements (onset at ~3 s after chair rotation). For all subjects, at peak SPV, the difference in amplitude between right and left gaze (ΔSPV = 20.4°/s) was significant for horizontal PRN (*p* = 0.0065) in the head upright paradigm (Figure 1A) and the TcNI was low (1.1 s, Table 1). When TcNI began to recover (TcNI > 5 s), the SPV of PRN had diminished into the range of 6–18°/s (mean 12°/s) and AL was no longer present despite the residual PRN.

**Table 1:** SPV, TcVOR, and TcNI for all three conditions.

**SCC stimulation and target configuration**	***n***	**Gaze**	**Peak SPV amplitude (°/s)**	**TcVOR (s)**	***P*-value right vs. left TcVOR**	**ΔSPV (°/s) at peak SPV (*p*-value)**	**TcNI (s) at peak SPV**
Horizontal (Figure 1A)	9	Right	60.6	9.9	0.4141	20.4 (0.00655)	1.1
		Left	81.0	11.0			
Combined Horizontal/Vertical (Figure 1B)	8	Right	55.6	8.8	0.948	18.7 (0.00882)	1.3
		Left	74.3	8.9			
Combined Horizontal/Vertical (Figure 1C)	6	Down	48.7	8.1	0.257	20.3 (0.00303)	1.0
		Up	69.0	10.7			

In the head tilted paradigm (combined horizontal/vertical SCC stimulation, Figure 1B) for all subjects the difference in the amplitude of the horizontal eye component of PRN (ΔSPV) between right and left gaze at peak SPV was 18.7°/s (*p* = 0.00882) corresponding to a TcNI of 1.3 s. Examining the vertical component in the head tilted paradigm (Figure 1C), the ΔSPV between up and down gaze at peak SPV was 20.3°/s (*p* = 0.00303) corresponding to a TcNI of 1.0 s. There was, however, no significant difference for the vertical component of the PRN at peak SPV (3 s time interval) in left or right gaze (*p* = 0.87). Likewise, there was no significant difference in the time constant of the decay (TcVOR) (*p* = 0.26) of PRN in up- and downward gaze.

**Figure 3:**
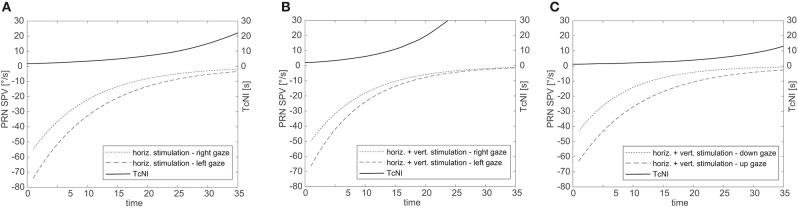
Aggregate results for all subjects for SPV decay over time for right and left gaze **(A,B)** and up and down gaze **(C)**. The solid line illustrates the corresponding TcNI. Note that the TcNI begins to return toward normal when SPV drop to the range of 8–16°/s.

Figure 3 illustrates the fitted negative exponential curves from all subjects derived from the parameters of the mixed effects model and the corresponding time constants (TcNI) for horizontal and combined horizontal and vertical SCC stimulations. Table 1 shows the aggregate results of all three tested conditions using the mixed effects model. There is statistically no difference in the TcVOR in any condition between left and right gaze or up and down gaze (range 8.1–11 s). In other words, TcVOR, reflected in the decay of PRN was not different between head orientations or eye positions in the orbit.

**Figure 4:**
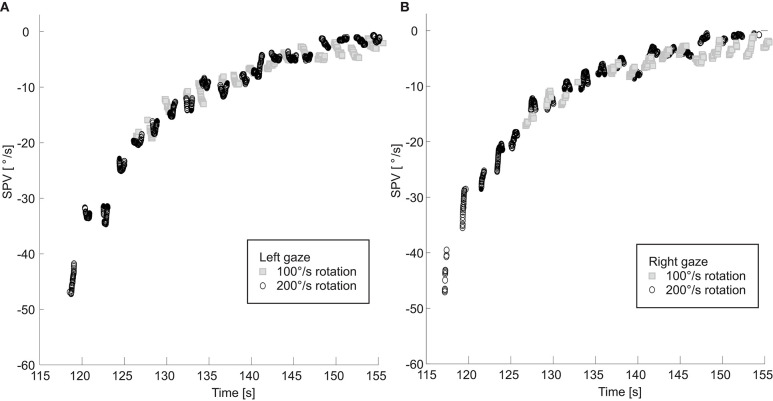
SPV is shown for rotations at 100 and 200°/s with right **(A)** and left **(B)** gaze. Note the two right and left SPV curves still overlap even when the initial slow phase velocities are different.

For one subject, we rotated the subject head upright at two different chair rotation velocities, 100°/s and 200°/s (Figures 4A,B). The two SPV curves overlapped, decaying at the same rate. In both paradigms the TcNI was initially decreased and then began to rise again when SPV dropped to comparable values: 21°/s at 3.5 s after peak velocity for the 100°/s rotation, vs. 18°/s at 12 s after peak velocity for the 200°/s rotation. In other words, the TcNI began to return toward normal when slow-phase velocity decreased to a range of values, rather than at the same time point in the decay of PRN.

## Discussion

The main findings of our study were 3-fold. AL was apparent almost immediately at the onset of PRN. AL began to fade when PRN reached a value in the 6–18°/s range. AL was present for pure horizontal and for mixed horizontal/vertical SCC stimulation. We will discuss possible mechanisms for these findings.

### Is AL an Adaptive Response?

Is AL a goal-driven, “*adaptive*” response to a *pathological* vestibular imbalance? The idea is that one can counteract the unwanted bias of a pathological SN by purposefully impairing the function of the ocular motor neural integrators (NI) that provide the signals to hold steady eccentric positions of gaze (2). By making the NI “leaky,” the nystagmus can be diminished or even nullified by moving the eyes to a position in the orbit in the direction of the slow phase of the SN. This concept reflects the general idea that the effect of persistent integration (perseveration) of unwanted biological noise—in this case the sustained pathological bias of SN—can be lessened by disabling neural integrator circuits. An analogous argument can be made for the gradual decrease (habituation) of the time constant of the velocity-storage integrator of the VOR when faced with the recurring nystagmus produced with repetitive constant-velocity (low frequency) chair rotations (2). The repetitive per- and post-rotatory nystagmus induced by this paradigm is interpreted as arising from a lesion, and hence the integrating (perseverating) circuits are disabled. Our results, however, suggest that the change in the TcNI was not time dependent. If it were adaptive it would more likely be time dependent.

### Is AL a Result of the Non-linear Properties of Neuronal Discharge Rates in the Vestibular Nuclei?–Results in Normal Subjects With Normal Patterns of Stimulation

The alternative hypothesis suggests that AL develops from the inherent physiological properties of the brainstem nuclei that process information both for the vestibulo-ocular reflex and for normal gaze holding. Particularly cogent arguments have been made for this view by the Zurich group (7, 11) and our results are largely in accord with their interpretation. First, using the decaying response of PRN as was induced in our rotatory chair paradigm, we confirmed that AL occurs in normal individuals, and when there is a natural reciprocal pattern of stimulation of both labyrinths. AL does not require that the sustained nystagmus be pathological as in a patient with loss of function in one labyrinth, nor produced with an unnatural stimulus in a normal individual as occurs with unilateral caloric stimulation. Future studies should investigate AL behavior in patients with unilateral vestibular loss or with vestibulocerebellar lesions.

### Does AL Take Time to Develop?

Within the limits of the capabilities of our experimental paradigms, AL appeared to begin promptly after the PRN began. There certainly could not have been a delay of more than a few seconds after the slow-phase velocity of the PRN reached its peak. We also showed a similar delay in the several subjects in whom we analyzed the earliest stage of their per-rotatory nystagmus. AL was already apparent once slow-phase velocity had reached its peak indicating that the prompt appearance of AL in the post-rotatory phase was not a result of the immediately preceding per-rotatory stimulus. Furthermore, we showed that the dissipation of AL as the PRN lessened was related to dropping to a specific range of values of slow-phase velocity (discussed further below), rather than the specific time during the decay of the PRN. In other words, we found no evidence of a delay in when AL is implemented by the brain once a substantial spontaneous nystagmus appeared, nor any relation of the AL effect to the duration of PRN.

On the other hand, our results do not exclude that other parameters of stimulation, such as the frequency content of the stimulus, the degree of eccentricity of eye positions during vestibular stimulation or the values of head acceleration or head velocity, influence the implementation of AL. For example, Anagnostou and Anastasopoulos (12, 13) showed no AL effect in normal subjects during horizontal or vertical head impulse testing (which is a high frequency, high acceleration stimulus). Similarly, Robinson et al. showed no AL effect for natural sinusoidal rotations (0.5 Hz) but the velocities were relatively low (peak velocity < 30°/s) (2).

### Is AL Related to the Amplitude of the Velocity Bias Created by a Sustained Nystagmus?

We showed a clear lessening of the AL effect over time in individual trials when the overall SPV of the PRN declined to a specific range of values. For all subjects at a mean value of 12.1°/s (range 6.4–18.1°/s) the inferred calculation of the TcNI increased relatively abruptly and soon reached a plateau at its previous normal high value. This finding is in accord with the result shown in Figure 4C of Bockisch et al. (14) in which the AL effect was greater for a higher SPV. This can be interpreted as the higher the SPV, the more likely the circuits that implement the VOR will show the non-linear effects of saturation, in which (1) neurons lose their ability to fire at higher rates in their excitatory direction and, (2) because of the effects of inhibitory cutoff at higher speeds of rotation, neurons can no longer decrease their discharge in a reciprocal fashion. The values for cutoff may be less in patients with unilateral hypofunction (15) since in these situations AL appears for even lower velocities of SN than we found in our normal subjects in our rotational chair paradigm.

Khojasteh et al. (11) developed a control systems model based on the known physiology of vestibular neurons in the face of asymmetrical inputs, and was able to simulate much of the known empirical data about AL. It is important to remember that the gaze-holding networks of the ocular motor NI for control of eye position are closely intertwined with the networks that generate the slow-phase velocities of the VOR. In the case of horizontal eye movements, for example, both functions are accomplished by shared neurons in the medial vestibular nucleus and the nucleus pre-positus hypoglossi (16). The saturation or inhibition effects of a high-velocity vestibular imbalance can be reflected in the effects of eye position on slow-phase velocity of the nystagmus, i.e., AL. In other words, as in the formulation of Khojasteh et al. (11), AL does not reflect changes in the function of the NI *per se*, but are driven by the direct effects of a vestibular imbalance on the same neurons in the vestibular nuclei that generate both the VOR and gaze-holding commands. Thus, neither a bias alone (e.g., Doslak et al. (6) and Jeffcoat et al. (8)) nor a change in the integrator alone (e.g., Robinson et al. (2)) explains AL.

As another result of our experiments, we noted that the AL effects were similar following both stimulation of the lateral SCC alone and of the lateral and vertical SCC together. The interaction between simultaneous horizontal and vertical stimulation had no impact on the AL effect nor on the overall TcVOR of either the horizontal or the vertical components of the PRN. Likewise, we showed that the AL effect was not directly related to vertical vs. horizontal eye positions in the orbit but rather to the direction of the vertical and horizontal eye movements produced relative to the requirements dictated by the pattern of stimulation of the SCC (17).

### Caveats and Limitations

We cannot exclude a small delay in the onset of AL, because our paradigm led to a strong nystagmus at the onset and offset of chair rotation, often causing artifacts due to blinks or imprecise gaze directions and thus decreased the amount of usable early data. Nystagmus might have been partially suppressed due to fixation on the blinking LEDs (20 ms) and potentially led to a smaller AL effect overall or at different times in the period of fading nystagmus. However, the relatively high speed of the nystagmus and the extremely brief period of exposure (20 ms) to the LED would lead to only a small amount of retinal image motion, equivalent to just a few degrees every 2 s that would not effectively drive visual suppression of the PRN.

Small suppression effects by head pitched downwards might have biased our results, however, these effects were considered negligible at 30° tilt compared to the traditional tilt suppression test with 90° head tilt (18).

The eccentric gaze positions and the timing of changing them in our paradigms were limited and symmetric. It is possible that our results would have been different if we used larger or smaller eccentric eye positions or different patterns of the timing of change in position. For example, effects like those that underlie rebound nystagmus might have influenced the estimates of the time constant of the neural integrator if there had been some asymmetry in the eccentric eye positions (19). In addition, we never measured the influence of AL on other aspects of function of the NI such as its direct effects on the phase of the VOR (20). More scrutiny of both the rise and the decay patterns of per- and post-rotatory nystagmus with a more sensitive recording technique such as with scleral search coils might reveal subtle deviations from the expected pattern from a simple exponential decay, and a more precise measure of the thresholds of the appearance and decay of AL. While the choice of the threshold when the TcNI began to recover was qualitative and by visual inspection the results were consistent among subjects.

## Conclusions

Even with its limitations our results strongly support the idea that AL develops because of the effects of the non-linear discharge properties of neurons within the common circuits that mediate the horizontal VOR and horizontal gaze-holding. Furthermore, stimulation of both vertical and horizontal SCC showed that AL was related to the predicted plane of rotation of the eyes based on the pattern of activation of the SCC.

## Data Availability Statement

The original contributions presented in the study are included in the article/supplementary materials, further inquiries can be directed to the corresponding author.

## Ethics Statement

The studies involving human participants were reviewed and approved by Kantonale Ethikkommission, KEK-Gesuchs-Nr.: 047/14, PB_2016-00680. The patients/participants provided their written informed consent to participate in this study.

## Author Contributions

CL: subject recruitment, data collection, data analysis, and drafting the article. DZ: conceptualization, supervision, methodology, data analysis, and drafting the article. TW: software, data analysis, and critical revision of the article. WW: data analysis and critical revision of the article. AK: data collection, database setup, and critical revision of the article. CS: database setup and critical revision of the article. MC: supervision, funding acquisition, and critical revision of the article. DZ, TW, WW, AK, CS, and MC: final approval of the version to be published. GM: conceptualization, supervision, methodology, data analysis, drafting the article, supervision, project administration, and funding acquisition. All authors contributed to the article and approved the submitted version.

## Conflict of Interest

The authors declare that the research was conducted in the absence of any commercial or financial relationships that could be construed as a potential conflict of interest.

## References

[1] Alexander G Die Ohrenkrankheiten im Kindesalter. In: Pfaundler M Schlossmann A, editors. Handbuch der Kinderheilkunge. Vogel: Leipzig (1912). p. 84–96.

[2] Robinson DA Zee DS Hain TC Holmes A Rosenberg LF. Alexander's law: its behavior and origin in the human vestibulo-ocular reflex. Ann Neurol. (1984) 16:714–22. 10.1002/ana.410160614 6441510

[3] Robinson DA. Eye movement control in primates. Science. (1968) 161:1219–24. 10.1126/science.161.3847.1219 5302604

[4] Bertolini G Tarnutzer AA Olasagasti I Khojasteh E Weber KP Bockisch CJ. Gaze holding in healthy subjects. PLoS ONE. (2013) 8:e61389. 10.1371/journal.pone.0061389 23637824 PMC3637181

[5] Hess K Reisine H. Counterdrifting of the eyes: additional findings and hypothesis. ORL J Otorhinolaryngol Relat Spec. (1984) 46:1–6. 10.1159/000275677 6608079

[6] Doslak MJ Dell'Osso LF Daroff RB. A model of Alexander's law of vestibular nystagmus. Biol Cybern. (1979) 34:181–6. 10.1007/BF00336969 314822

[7] Khojasteh E Bockisch CJ Straumann D Hegemann SC. A dynamic model for eye-position-dependence of spontaneous nystagmus in acute unilateral vestibular deficit (Alexander's Law). Eur J Neurosci. (2013) 37:141–9. 10.1111/ejn.12030 23106392

[8] Jeffcoat B Shelukhin A Fong A Mustain W Zhou W. Alexander's law revisited. J Neurophysiol. (2008) 100:154–9. 10.1152/jn.00055.2008 18450584

[9] Holm S A simple sequentially rejective multiple test procedure. Scand J Stat. (1979) 6:65–70.

[10] R Core Team A Language and Environment for Statistical Computing. Vienna: R Foundation for Statistical Computing (2018).

[11] Khojasteh E Bockisch CJ Straumann D Hegemann SC. A mechanism for eye position effects on spontaneous nystagmus. Conf Proc IEEE Eng Med Biol Soc. (2012) 2012:3572–5. 10.1109/EMBC.2012.6346738 23366699

[12] Anastasopoulos D Anagnostou E. Invariance of vestibulo-ocular reflex gain to head impulses in pitch at different initial eye-in-orbit elevations: implications for Alexander's law. Acta Otolaryngol. (2012) 132:1066–72. 10.3109/00016489.2012.682120 22668130

[13] Anagnostou E Heimberger J Sklavos S Anastasopoulos D. Alexander's law during high-acceleration head rotations in humans. Neuroreport. (2011) 22:239–43. 10.1097/WNR.0b013e3283451769 21346643

[14] Bockisch CJ Khojasteh E Straumann D Hegemann SCA. Eye position dependency of nystagmus during constant vestibular stimulation. Exp Brain Res. (2013) 226:175–82. 10.1007/s00221-013-3423-6 23386125

[15] Hegemann S Straumann D Bockisch C. Alexander's law in patients with acute vestibular tone asymmetry–evidence for multiple horizontal neural integrators. J Assoc Res Otolaryngol. (2007) 8:551–61. 10.1007/s10162-007-0095-6 17879115 PMC2538344

[16] Cannon SC Robinson DA. Loss of the neural integrator of the oculomotor system from brain stem lesions in monkey. J Neurophysiol. (1987) 57:1383–409. 10.1152/jn.1987.57.5.1383 3585473

[17] Bockisch CJ Hegemann S. Alexander's law and the oculomotor neural integrator: three-dimensional eye velocity in patients with an acute vestibular asymmetry. J Neurophysiol. (2008) 100:3105–16. 10.1152/jn.90381.2008 18799600

[18] Hain TC Zee DS Maria BL. Tilt suppression of vestibulo-ocular reflex in patients with cerebellar lesions. Acta Otolaryngol. (1988) 105:13–20. 10.3109/00016488809119440 3341153

[19] Otero-Millan J Colpak AI Kheradmand A Zee DS. Rebound nystagmus, a window into the oculomotor integrator. Prog Brain Res. (2019) 249:197–209. 10.1016/bs.pbr.2019.04.040 31325980

[20] Skavenski AA Robinson DA. Role of abducens neurons in vestibuloocular reflex. J Neurophysiol. (1973) 36:724–38. 10.1152/jn.1973.36.4.724 4197340

